# Changes in Thyroid Hormone Signaling Mediate Cardiac Dysfunction in the Tg197 Mouse Model of Arthritis: Potential Therapeutic Implications

**DOI:** 10.3390/jcm10235512

**Published:** 2021-11-25

**Authors:** Lydia Ntari, Polyxeni Mantzouratou, Athanasia Katsaouni, Constantinos Pantos, George Kollias, Iordanis Mourouzis

**Affiliations:** 1 Biomedcode Hellas SA, 34 Fleming Street, 16672 Vari, Greece; lydia.ntari@gmail.com; 2 Department of Pharmacology, School of Medicine, National and Kapodistrian University of Athens, 11527 Athens, Greece; polyxenis@gmail.com (P.M.); athanasia.paraskevikatsaouni@gmail.com (A.K.); cpantos@med.uoa.gr (C.P.); 3 Biomedical Sciences Research Center “Alexander Fleming” (BSRC Fleming), Institute for Bioinnovation, 16672 Vari, Greece; kollias@fleming.gr; 4 Department of Physiology, School of Medicine, National and Kapodistrian University of Athens, 11527 Athens, Greece

**Keywords:** cardiac dysfunction, heart failure, arthritis, thyroid hormone, thyroid hormone receptors

## Abstract

Background Rheumatoid Arthritis (RA) patients show a higher risk of heart failure. The present study investigated possible causes of cardiac dysfunction related to thyroid hormone (TH) signaling in a RA mouse model. Methods A TNF-driven mouse model of RA[TghuTNF (Tg197)] was used. Cardiac function was evaluated by echocardiography. SERCA2a and phospholamban protein levels in left ventricle (LV) tissue, thyroid hormone levels in serum, TH receptors in LV and TH-related kinase signaling pathways were measured. T3 hormone was administered in female Tg197 mice. Results We show LV and atrial dilatation with systolic dysfunction in Tg197 animals, accompanied by downregulated SERCA2a. We suggest an interaction of pro-inflammatory and thyroid hormone signaling indicated by increased p38 MAPK and downregulation of TRβ1 receptor in Tg197 hearts. Interestingly, female Tg197 mice showed a worse cardiac phenotype related to reduced T3 levels and Akt activation. T3 supplementation increased Akt activation, restored SERCA2a expression and improved cardiac function in female Tg197 mice. Conclusions TNF overexpression of Tg197 mice results in cardiac dysfunction via p38 MAPK activation and downregulation of TRβ1. Gender-specific reduction in T3 levels could cause the worse cardiac phenotype observed in female mice, while T3 administration improves cardiac function and calcium handling via modified Akt activation.

## 1. Introduction

Chronic Inflammatory Joint diseases, such as Rheumatoid arthritis (RA), Spondyloarthritis and Psoriatic Arthritis are mainly associated with articular inflammation eventually leading to bone erosion and thus joint damage. However, the high morbidity rate observed in these patients is mainly related to the high incidence of disease affecting certain extra-articular organs [1]. Cardiovascular diseases (CVD), including ischemic heart disease, heart failure (HF) and valvular heart disease (VHD) are the most common co-morbidities of RA patients reaching up to 70–80% incidence [2], while RA is considered as an independent risk factor for cardiovascular disease [3]. The incidence of HF in RA patients is 2-fold higher [4,5] being a major cause of their premature mortality [6]. The prevalence of HF in RA is high even after adjusting for ischemic heart disease indicating the existence of mechanisms beyond atherosclerosis and traditional risk factors [3,7]. In accordance, recent experimental evidence supports a link between systemic inflammation in arthritis animal models and cardiac dysfunction [8,9]. However, more mechanistic studies are needed to elucidate the underlying mechanisms. Unravelling these mechanisms would allow a better understanding of the pathophysiology of HF in RA patients, facilitating the development of new and more specific treatments. In this regard, we have previously showed that the human Tumor Necrosis Factor (huTNF) Tg197 arthritis mouse model [10], in parallel to its RA pathology, also develops TNF-driven VHD, which mainly leads to valvular thickening with some degree of stenosis and occasionally insufficiency [11]. Beyond VHD, pro-inflammatory signals may directly provoke cardiac dysfunction via activation of Mitogen Activated Protein Kinases (MAPKs), such as p38 MAPK and c-Jun N-terminal kinases (JNKs), [12] or dysregulate other neuroendocrine systems important in HF development [13]. In fact, TNF signaling has been shown to interact with the thyroid hormone signaling pathway [14,15] which has been increasingly recognized to play a critical role in cardiac dysfunction [16]. Thyroid hormone receptors and their natural ligand (triiodothyronine, T3) regulate important kinase signaling pathways (such as ERK and AKT) as well as contractile and calcium handling proteins in the myocardium [17]. As a result, thyroid hormone levels, even within normal range are strictly related to cardiac function [18]. Thus, the present study investigated novel underlying mechanisms of cardiac dysfunction related to TH signaling in both genders of an RA mouse model [TghuTNF (Tg197)]. Gender is an important determinant of cardiovascular risk in RA patients, with HF and premature CVD mortality being much higher in women than men [19,20].

## 2. Methods

### 2.1. Mice

This investigation conforms to the Guide for the Care and Use of Laboratory Animals published by the US National Institutes of Health (NIH publication No 85-23, revised 1985). The protocol of the study was approved by the Animal Care and Use Committee of Department of Pharmacology, Medical School, National and Kapodistrian University of Athens (license 6105/16 November 2017, ΕL 25BIOexp 10) and has therefore been performed in accordance with the ethical standards laid down in the 1964 Declaration of Helsinki and its later amendments. Tg197 mice were provided by the animal facilities of Biomedical Sciences Research Center (BSRC) “Alexander Fleming” and have been previously described [21]. Mice were maintained on a C57BL/6JxCBA genetic background. The animal facility of Department of Pharmacology, Medical School, NKUA was under a 12:12-h light/dark cycle at a constant temperature of 22 ± 1.5 °C and relative humidity of ~50%, under SPF conditions. All mice were handled according to the guidance of the Institutional Animal Care and Use Committee of National and Kapodistrian University of Athens. All mice were observed for morbidity and euthanized when needed according to animal welfare guidelines. All animals were sacrificed at 11–12 weeks of age.

### 2.2. Experimental Protocol

The following group of animals were studied:

1. Male WT C57BL/6JxCBA mice at 11–12 weeks of age, *n* = 14
2. Male Tg197 mice at 11–12 weeks of age, *n* = 13
3. Female WT C57BL/6JxCBA mice at 11–12 weeks of age, *n* = 14
4. Female Tg197 mice at 11–12 weeks of age, *n* = 16

After echocardiography, mice were sacrificed, the heart was excised and the left ventricle was frozen in liquid nitrogen for further molecular analysis. Blood was collected, centrifuged and serum was kept at −80 °C for determination of thyroid hormone levels. In order to investigate the role of T3 deficiency in aggravating contractile dysfunction in female Tg197 mice, 3,5,3′-triiodothyronine was administered subcutaneously in a replacement dose for 10 days from 10th to 11th week (0.13 μg/g every 48 h, TgFem + Τ3, *n* = 6). Another group of female Tg197 mice received subcutaneous injections of vehicle (TgFem + Placebo, *n* = 7).

### 2.3. Echocardiography

Echocardiography assessment was performed in the Department of Pharmacology, Medical School NKUA, Greece as previously described [22]. Briefly, mice were sedated with intraperitoneal injection of ketamine-midazolam cocktail and were placed on a heated platform to maintain the body temperature at 37 °C. Echocardiographic images in parasternal short and long axes were acquired using a Vivid 7 version Pro ultrasound system (GE Healthcare, Wauwatosa, WI, USA), equipped with a 14.0-MHz probe (i13L) in a blinded to the genotype fashion.

### 2.4. Measurement of Thyroid Hormones

Plasma L-thyroxine (T4) and 3,5,3′ tri-iodothyronine (T3) quantitative measurements were performed with EIAT3C T3andEIAT4CT4ELISA kits (ThermoScientific LSG, MA, USA), according to manufacturer’s instructions. L-thyroxine and 3,5,3′ triiodothyronine levels were expressed as ng/mL of plasma. Measurements were performed with Genios ELISA reader (Tecan, Männedorf, Switzerland).

### 2.5. Administration of Triiodothyronine

T3 (Sigma Chemicals, St Louis, MO, USA) was dissolved in ethanol by adding a small volume of 25% NaOH and diluted in 0.9% normal saline to obtain a stock solution with a concentration of 1 mg/mL. Stock solution was kept at −20 °C and before each experiment a quantity of this solution was dissolved in normal saline at the final concentration of 50 μg/mL and was administered subcutaneously at a dose of 0.13 μg/g every 48 h for 10 days. The control group was treated with vehicle in a similar manner.

### 2.6. Protein Isolation, Sodium Dodecyl Sulfate-Protein Polyacrylamide (SDS-PAGE) Gel Electrophoresis and Immunodetection

Left ventricular tissue was homogenized and subjected to the previously described procedure to isolate cytosolic and nuclear fraction. TRs protein expression was determined in nuclear fraction, while SERCA, phospholamban and kinases were determined in cytosolic fraction. Protein concentrations were determined by the BCA method.

Samples were prepared for sodium dodecyl sulfate polyacrylamide gel electrophoresis (SDS-PAGE) and Western blotting as previously described [22,23]. After Western blotting, filters were probed with specific antibodies against TRα1 (ab53729, Abcam, Cambridge, United Kingdom), TRβ1 (MA1-216, Affinity BioReagents, Colorado, USA), histone H3 (#9715, Cell Signaling Technology, MA, USA), SERCA (MA3-919, Affinity BioReagents), phospholamban (MA3-922, Affinity BioReagents), total and phosphorylated–ERK, (p-ERK) (#9101, #9102, Cell Signaling Technology), total and phosphorylated-Akt, (p-Akt) (#9271, #9272, Cell Signaling Technology), total and phosphorylated-p38 MAPK (#9211, #9212, Cell Signaling Technology), total and phosphorylated-JNKs (#9251, #9252, Cell Signaling Technology), and actin (A5316, Sigma Aldrich, MO, USA) all at concentration 1:1000 and overnight incubation at 4 °C. Filters were incubated with appropriate anti-mouse (Amersham Biosciences, Amersham, United Kingdom) or anti-rabbit (Cell Signaling Technology) HRP secondary antibodies. Immunoreactivity was detected by enhanced chemiluminescence using Lumiglo reagents (New England Biolabs, MA, USA). Chemiluminescence was detected by the digital image analysis system FluorChem HD2 (Alpha Innotech Corporation, San Leandro, CA, USA) equipped with a CCD camera and analysis software. Results were expressed as the ratio of phosphorylated kinase levels to total kinase levels. Histone H3 or actin protein expression was used in order to normalize slight variations in protein loading [22].

### 2.7. Statistical Analysis

Results are presented as mean ± SEM. One-way analysis of variance with Bonferroni or Dunnett correction was used for multiple comparisons. Student’s t test or Mann-Whitney test was used to evaluate differences between groups concerning data from protein analysis. Two-way ANOVA with Fischer’s Least Significant Difference (LSD) post hoc test was used in analysis of echocardiography data in order to evaluate the changes in Tg197 mice versus WT and the effect of gender (male vs. female). Significance was set at 0.05. Analysis was performed using the SPSS 23.0 software (IBM Corp., Armonk, NY, USA).

## 3. Results

### 3.1. Development of Left Ventricular Dysfunction in the Tg197 Arthritis Model in Both Genders

As we have recently shown that Tg197 animals develop spontaneous left ventricular dysfunction [11], we sought to investigate further the cardiac phenotype of these mice and any gender-dependent differences that may appear. For this reason, we performed extensive echocardiographic assessment in Tg197 and WT males and females in their 11th week of age. Echocardiographic assessment revealed that left ventricular internal diameter at end-diastolic phase (LVEDd) and left ventricular internal diameter at end-systolic phase (LVESd) were significantly increased in both Tg197 males and females compared to WT mice (Table 1). Furthermore, left atrial (LA) diameter, an indicator of LV filling pressure, as well as the thickness of the posterior wall of LV (LVPWd) were also found to be significantly increased in Tg197 males and females compared to WT mice, indicating cardiac hypertrophy. Indices of LV function, such as ejection fraction and systolic velocity of posterior wall thickening (SVPW), were significantly reduced in Tg197 males and females compared to WT mice, indicating global LV and regional contractile dysfunction respectively (Table 1).

Interestingly, female Tg197 mice showed increased cardiac dysfunction compared to Tg197 male mice. More specifically, LVESd, LA diameter, LVPWd and SVPW were significantly increased in females compared to male Tg197 animals (Two-way ANOVA between genotype and gender F = 4.6, *p* = 0.036 for LVESd, F = 24.3, *p* = 0.00008 for LA, F = 4.2, *p* = 0.045 for LVPWd and F = 4.7, *p* = 0.036 for SVPW). However, ejection fraction did not differ between male and female Tg197 animals.

### 3.2. Downregulation of Calcium Cycling Protein Expression in LV of Tg197 Mice Reveals Cardiac Remodeling at the Molecular Level

**Figure 1:**
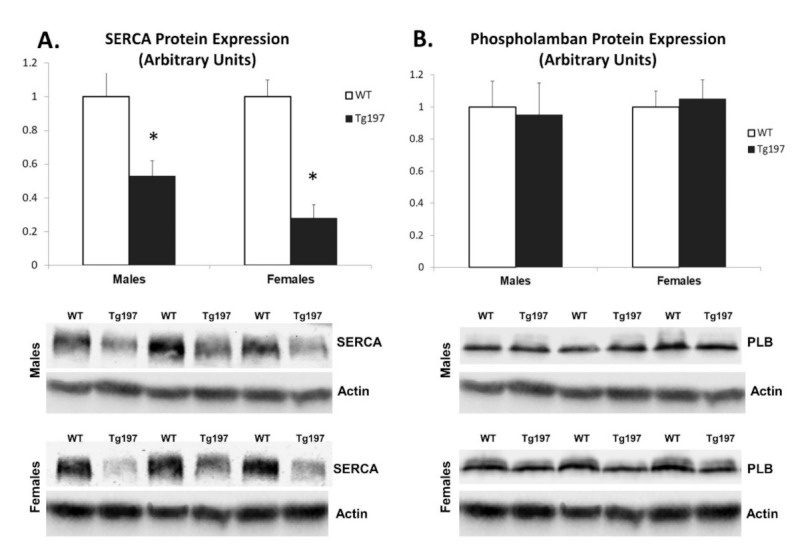

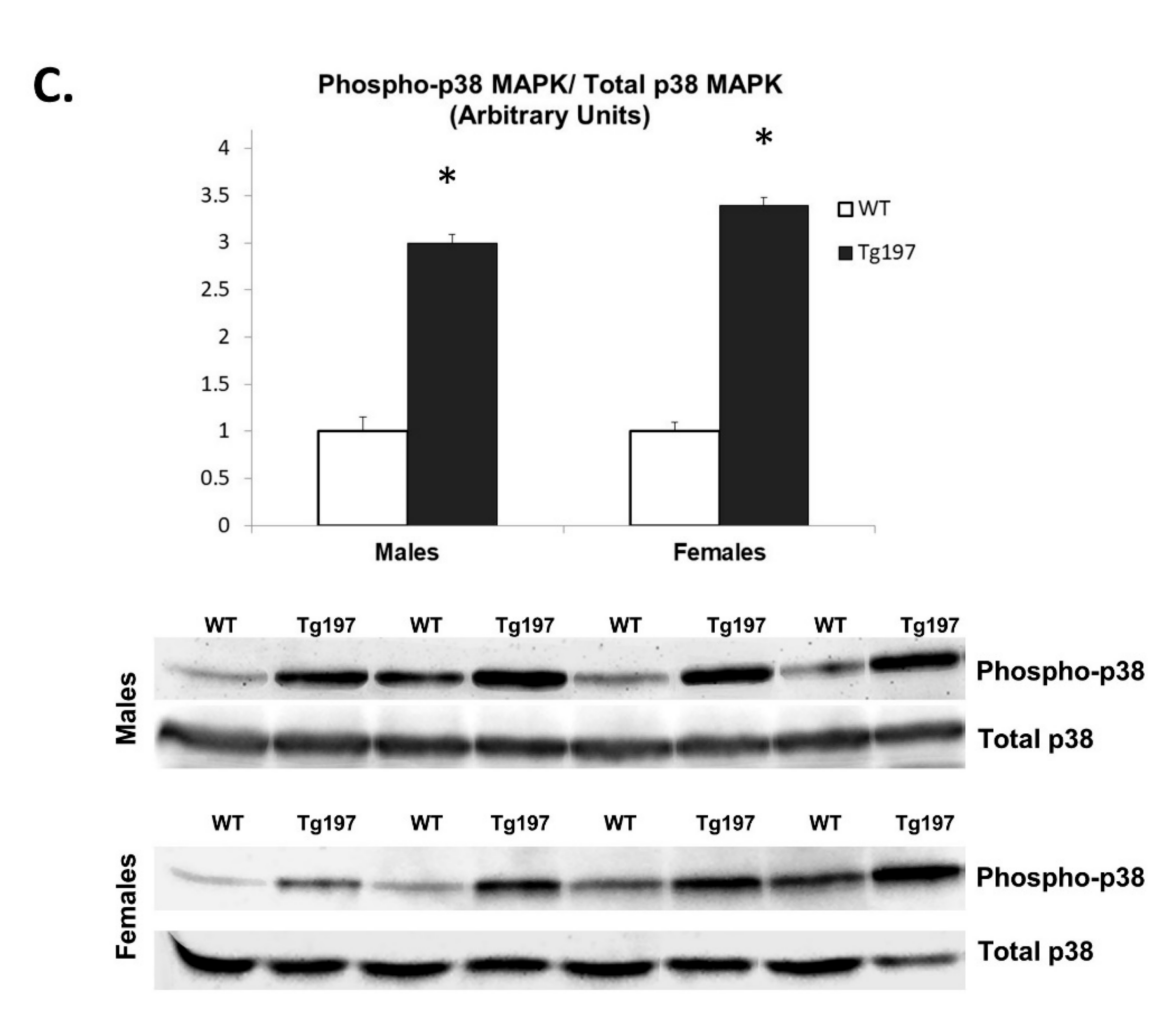
Reduced SERCA and increased activation of p38 MAPK in Tg197 LV samples. Densitometric assessment in arbitrary units and representative images of SERCA2a protein expression (**A**), PLB protein expression (**B**) and phosphorylated and total p38 MAPK (**C**) in left ventricle of WT C57BL/6JxCBA (WT, *n* = 4 for each gender) and Tg197 (*n* = 6 for each gender) male and female mice. Values are represented in mean ± SEM; * *p* < 0.05. (SERCA: Sarcoplasmic Reticulum–Calcium ATPase, PLB: Phospholamban, LV: left ventricle).

Downregulation of SERCA2a and/or upregulation of phospholamban lead to severe impairment of calcium handling and contractile function serving as a hallmark of cardiac remodeling and heart failure [24]. Interestingly, expression of SERCA protein in LV tissue of Tg197 males in their 11th week of age was found to be significantly reduced by 2-fold compared to WT males, while it was reduced 3.6-fold in Tg197 female mice compared to WT females. On the other hand, phospholamban protein expression did not differ between Tg197 and WT mice either in males or in females (Figure 1A,B). These results indicate heart failure specific molecular changes in cardiomyocytes in Tg197 mice.

### 3.3. Activation of Pro-Inflammatory Kinase Signaling in Myocardium of Tg197 Animals in Both Genders

Pro-inflammatory signals have been shown to activate Mitogen Activated Protein Kinases (MAPKs), such as p38 MAPK and JNK, which are known to exert a critical role in cardiac dysfunction and HF [12]. Thus, we investigated whether JNK and/or p38 MAPK signaling is activated in the LV tissue of Tg197 animals. Although the ratio of phosphorylated p54 JNK to total p54 JNK in myocardium of Tg197 animals was similar to WT animals both in male and female mice, the ratio of phosphorylated p38 MAPK to total p38 MAPK was found increased by 3.0-fold in male Tg197 and by-3.4 fold in female Tg197 compared to WT male and female samples, respectively (Figure 1C).

### 3.4. Changes in Thyroid Hormone Signaling Pathway in Myocardium of Tg197 Animals in Both Genders

**Figure 2:**
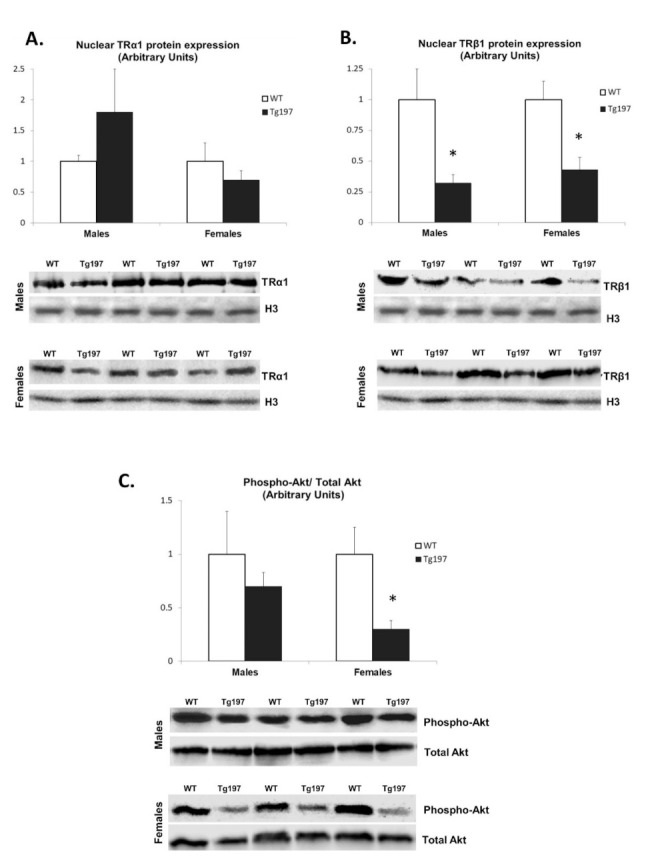
Reduction of TRβ1 in Tg197 LV samples and female-specific reduction of Akt activation. Densitometric assessment in arbitrary units and representative Western blots of thyroid hormone receptor α1 nuclear expression (TRα1, **A**), thyroid hormone receptor β1 nuclear expression (TRβ1, **B**) and phosphorylated and total p38 MAPK (**C**) in left ventricle of WTC57BL/6JxCBA (WT, *n* = 4 per gender) and Tg197 (*n* = 6 per gender) male and female mice. Values are represented in mean ± SEM; * *p* < 0.05 (H3: Histone 3).

Pro-inflammatory signals have been shown to interact with other important signaling pathways related to HF, such as the thyroid hormone signaling pathways [14]. Therefore, we investigated whether TR expression is altered in Tg197 heart tissues. Nuclear expression of TRα1 was not found to be significantly different between Tg197 and WT mice either in males or in females (Figure 2A). However, nuclear expression of TRβ1 was found to be significantly reduced by 3-fold and 2.3-fold in LV tissue derived from Tg197 male and female mice respectively compared to their WT littermates (Figure 2B). As TRβ1 is known to regulate SERCA gene expression [23,24], our findings strongly suggest the involvement of TRβ1 in down-regulation of SERCA and cardiac dysfunction in Tg197 hearts.

Additionally, we studied thyroid hormone levels in serum (T4 and T3), which are the natural ligands of TRs. T4 levels were not significantly different between Tg197 and WT mice (106 ± 10 ng/mL for WT vs. 74 ± 12 for Tg197 males and 111 ± 23 for Tg197 females, *p* > 0.05). Interestingly, T3 levels were found to be differentially affected only in Tg197 female mice. More specifically, levels of T3 in serum of female Tg197 mice were significantly decreased compared to WT female mice (694 ± 53 pg/mL for WT vs. 442 ± 97 for Tg197 females, *p* < 0.05), while T3 levels in male Tg197 animals did not differ compared to WT males (694 ± 53 pg/mL vs. 876 ± 106 pg/mL, *p* > 0.05). As a result, the ratio of T3 to T4 was significantly decreased by 110% in female Tg197 compared to male Tg197 and by 43% as compared to WT mice, *p* < 0.05.

### 3.5. Activation of TH-Related Kinase Signaling in Myocardium of Tg197 Animals in Both Genders

We examined TH-related ERK and Akt signaling activation in Tg197 animals [22]. In females, the ratio of phosphorylated Akt to total Akt was reduced by 3.9-fold in LV tissues of Tg197 compared to WT (Figure 2C). However, there was no significant difference in the ratio of phosphorylated Akt to total Akt in male mice. In addition, the ratio of phosphorylated p44 and p42 ERK to total ERK in both genders was similar. This evidence suggests that a gender-specific reduction in T3 levels could result in reduced Akt activation with important potential consequences on calcium handling and worse cardiac phenotype in female Tg197 mice.

### 3.6. Effect of T3 Administration in Tg197 Female Mice

**Figure 3:**
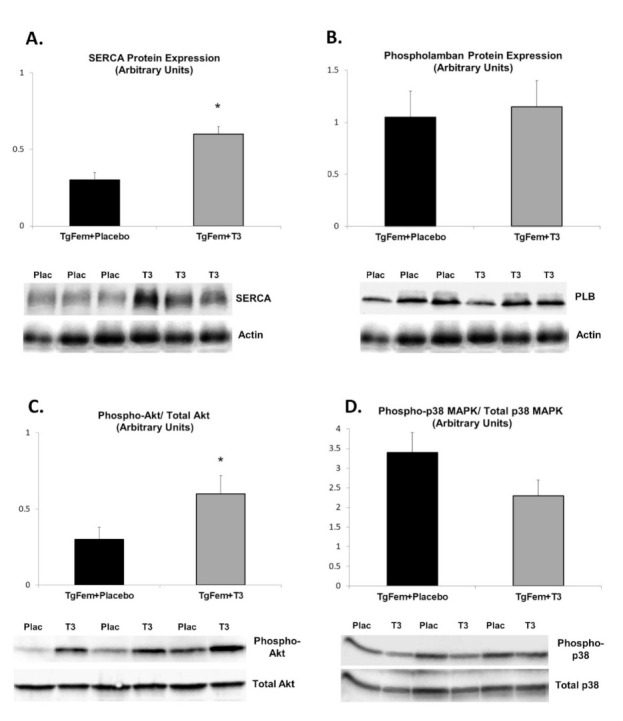
Reversal of molecular phenotype of cardiac dysfunction in Tg197 female mice upon treatment with T3. Densitometric assessment in arbitrary units and representative images of SERCA2a protein expression (**A**), PLB protein expression (**B**), phosphorylated and total Akt (**C**) and phosphorylated and total p38 MAPK (**D**) in left ventricle of TgFem + Placebo versus TgFem + Τ3 female mice. * *p* < 0.05. (SERCA: Sarcoplasmic Reticulum–Calcium ATPase, PLB: Phospholamban, LV: left ventricle).

In order to prove the role of T3 deficiency in Tg197 female mice, we administered T3 or vehicle for 10 days in a separate group of Tg197 female animals. Echocardiographic analysis revealed a significant improvement in cardiac contractile function in TgFem + T3 mice (Table 2). More specifically, LVESd and LA diameter were reduced while Ejection Fraction and SVPW were increased in TgFem + T3 as compared to TgFem + Placebo mice (Table 2). This improvement was accompanied by favorable changes in the expression of calcium handling proteins. Expression of SERCA protein in LV tissue of TgFem + T3 mice was found to be significantly increased by 2.0-fold compared to TgFem + Placebo, while phospholamban protein expression did not differ between the two groups (Figure 3A,B). Furthermore, the ratio of phosphorylated Akt to total Akt was increased by 2.0-fold in LV tissues of TgFem + T3 compared to TgFem + Placebo (Figure 3C). On the contrary, the ratio of phosphorylated p38 MAPK to total p38 MAPK was reduced by 1.33-fold in TgFem + T3 versus TgFem + Placebo mice, but this difference did not reach statistical significance (Figure 3D).

## 4. Discussion

In the present study, we show that the TghuTNF (Tg197) arthritis model, in addition to its chronic polyarthritis, also develops cardiac dysfunction. More specifically, our observations suggest development of cardiac remodeling and heart failure, mainly characterized by LV and LA dilatation, as well as diastolic and systolic dysfunction. These features are also observed in RA patients [2]. In fact, diastolic cardiac dysfunction is the initial presentation of 66% of RA patients before progression to overt HF [25]. In accordance with the echocardiographic results, we found that LV tissues of Tg197 mice express downregulated levels of SERCA2a protein, a molecular hallmark of both cardiac remodeling and HF [24,26]. This finding signifies severe impairment of calcium handling in the myocardium of Tg197 mice, as SERCA2a is predominantly expressed in cardiomyocytes regulating systolic and diastolic function [27]. As the main source of TNF in Tg197 animals is mesenchymal cells, our data suggest that the altered phenotype of cardiac mesenchymal fibroblasts [11] could regulate their local microenvironment and trigger the progression of HF via interaction with cardiomyocytes. In fact, stromal cells are now recognized as key mediators of various inflammatory diseases [28].

Pro-inflammatory signaling pathways have a critical role both in cardiac remodeling as well as in cartilage and bone destruction in RA via activation of MAPKs, such as p38 MAPK and JNK [12]. p38 MAPK has been also suggested to negatively regulate cardiac contractile function via changes in calcium handling [29]. We show here enhanced activation of p38 MAPK in heart tissues of Tg197 mice, which could contribute to SERCA downregulation [30] and hence cardiac dysfunction in the Tg197 animals.

Pro-inflammatory signals have also been shown to interact with other important neuroendocrine systems, such as the thyroid hormone [14,15] which has also been increasingly recognized to play a critical role in cardiac dysfunction [16]. Thyroid hormone receptors (TRs) and their natural ligand (T3) regulate important kinase signaling pathways, such as ERK and Akt, as well as contractile and calcium handling proteins in the myocardium [17]. TRα1 and TRβ1 are the most abundant in cardiac muscle and their downregulation has been linked to infarction-related HF, while treatment with thyroid hormone has been considered as a new therapeutic approach [31]. In the process of aging and age-related diseases, conversion of T4 to T3 is impaired, while the lower FT3/FT4 ratio has been associated with an impaired functional status (activities of Daily Living scale or the frailty index) and an increased mortality [32,33]. In addition, in synovial fibroblasts from RA patients a reduction in TRβ1 expression was recently found [15]. Interestingly, we show here a TRβ1downregulation in Tg197 animals, which indicates an involvement of TRβ1 in their cardiac dysfunction. As TRβ1, a transcription factor, is known to regulate SERCA gene expression [34], our findings strongly suggest a role of the TH system in downregulation of SERCA and cardiac dysfunction in Tg197 animals.

In clinical studies, gender is found to be an important determinant of cardiovascular risk in RA patients, with HF and premature CVD mortality being much higher in women than men [19,20]. In accordance with these findings, we showed enhanced cardiac dysfunction in female Tg197 hearts compared to males. In addition, our data indicate that deterioration of cardiac function was associated with a female-specific reduction in serum T3 levels. Interestingly, this finding agrees with recent clinical observations which suggest that female patients with RA have an increased incidence of thyroid disorders reaching up to 80% [35].

T3 has been previously shown to activate the PI3K/Akt pathway via the interaction of TRα1 with the p85α subunit of PI3K [36]. The regulatory action of Akt on calcium cycling proteins has been previously established in studies in animal models [37]. Therefore, we examined ERK and Akt signaling activation in hearts of Tg197 animals [22]. Akt activation was significantly decreased only in female Tg197 mice, further suggesting that gender-specific changes in T3 levels could result in reduced Akt activation and account for the worse cardiac phenotype observed in female Tg197 mice.

We have previously found that TH treatment in animal models of infarction-related HF results in activation of Akt, increased SERCA/phospholamban ratio and improved cardiac function [22]. We, therefore, treated Tg197 female mice with T3 for 10 days in a late stage of heart disease. Interestingly, only a short-period treatment with T3 was sufficient to improve LV function, resulting in increased global and regional contractile function and restoration of LV and LA dilation. Furthermore, this improvement was accompanied by favorable changes in the expression of SERCA and increased activation of Akt signaling pathway in T3-treated mice. These data show for the first time that treatment of T3 deficiency in female Tg197 mice improves cardiac dysfunction and calcium handling via Akt activation in RA-related heart disease.

## 5. Limitations and Future Perspectives

Although our findings point to new directions in the treatment of RA-related HF, results from animal models should be always interpreted with caution in clinical practice. In addition, the effect of T3 treatment in the arthritis pathology and prognosis of the disease remains unanswered as well as the effectiveness of T3 in preventing cardiac dysfunction if administered early during the course of the disease. Despite these limitations, the present study opens a new area of investigation as the role of thyroid hormone in RA-induced cardiac dysfunction has not been studied before. The role of T3 in RA-related heart pathology should be further investigated in additional arthritis models and in patients. Based on the above, clinical studies are needed to investigate the relationship between thyroid hormone levels even within normal range and cardiac function in RA patients. As specific treatments for cardiac dysfunction in RA do not exist, T3 supplementation could prove a novel therapeutic approach specifically targeting RA induced heart failure. The optimal dose and timing of administration could be the aim of future trials.

## 6. Conclusions

We showed that enhanced mesenchymal-specific TNF signaling can result in cardiac dysfunction and impaired calcium handling via p38 MAPK activation and downregulation of TRβ1 in the myocardium of Tg197 mice. In addition, gender-specific reduction in T3 levels may account for the worse cardiac phenotype observed in female Tg197 mice, while exogenous T3 supplementation improves cardiac function and calcium handling via modified Akt activation. These findings may be of high physiological and therapeutic relevance.

## Figures and Tables

**Table 1:** Echocardiographic analysis in male and female Tg197 and WT mice.

	Males			Females		
	WT (*n* = 14)	Tg197 (*n* = 13)	*p* Value for Males	WT (*n* = 14)	Tg197 (*n* = 16)	*p* Value for Females
Body weight, g	28.6 ± 0.7	19.9 ± 1.0	0.00001	22.10 ± 0.5	14.2 ± 0.4	10^−10^
LVEDd, mm/BW	0.13 ± 0.002	0.19 ± 0.008	0.0001	0.16 ± 0.004	0.23 ± 0.006	10^−9^
LVEDs, mm/BW	0.08 ± 0.003	0.12 ± 0.006	0.00004	0.09 ± 0.003	0.15 ± 0.006	10^−7^
LVPWd, mm/BW	0.024 ± 0.0007	0.034 ± 0.0018	0.001	0.028 ± 0.0010	0.044 ± 0.0014	10^−8^
LA, mm/BW	0.08 ± 0.002	0.11 ± 0.005	0.00005	0.09 ± 0.002	0.15 ± 0.003	10^−14^
SVPW, cm/s	3.05 ± 0.06	2.46 ± 0.07	10^−8^	2.84 ± 0.05	2.02 ± 0.03	10^−12^
Ejection Fraction (%)	74.6 ± 0.8	67.8 ± 1.8	0.02	73.7 ± 1.0	64.1 ± 1.5	0.0001

LVEDd: left ventricular end-diastolic diameter; LVEDs: left ventricular end-systolic diameter; LVPWd: left ventricular end-diastolic posterior wall thickness; LA: left atrium; SVPW: systolic velocity of the posterior wall. Values were normalized with the body weight (except for SVPW), as indicated in the table. Data were expressed as mean ± SEM. One-way analysis of variance with Bonferroni or Dunnett T3 correction was used for multiple comparisons. Two-way ANOVA was used to examine the interaction of both genotype (Tg197 or WT00) and gender on echocardiographic parameters. Two-way ANOVA showed a significant interaction between genotype and gender in LVEDs (F = 4.6, *p* = 0.036), LA (F = 24.3, *p* = 0.00008), LVPWd (F = 4.2, *p* = 0.045) and SVPW (F = 4.7, *p* = 0.036).

**Table 2:** Echocardiographic analysis in female Tg197 mice receiving either placebo or T3 for 10 days from 10th to 11th week.

	TgFem + Placebo (*n* = 7)	TgFem + T3 (*n* = 6)	*p* Value
Body weight, g	14.0 ± 0.6	14.2 ± 0.7	0.7
LVEDd, mm/BW	0.24 ± 0.009	0.22 ± 0.016	0.15
LVEDs, mm/BW	0.16 ± 0.01	0.11 ± 0.01	**0.01**
LVPWd, mm/BW	0.044 ± 0.0014	0.044 ± 0.0033	0.85
LA, mm/BW	0.15 ± 0.003	0.12 ± 0.006	**0.001**
SVPW, cm/s	2.02 ± 0.03	2.88 ± 0.12	**0.0001**
Ejection Fraction (%)	64.1 ± 1.5	80.2 ± 1.6	**0.0005**

LVEDd: left ventricular end-diastolic diameter; LVEDs: left ventricular end-systolic diameter; LVPWd: left ventricular end-diastolic posterior wall thickness; LA: left atrium; SVPW: systolic velocity of the posterior wall. Values were normalized with the body weight (except for SVPW), as indicated in the table. Data are expressed as mean ± SEM. Bold indicate the statistically different values with bold (*p* values < 0.05).

## Data Availability

Data are available upon request to the corresponding author.
